# LRCFuse: Infrared and Visible Image Fusion Based on Low-Rank Representation and Convolutional Sparse Learning

**DOI:** 10.3390/s26061771

**Published:** 2026-03-11

**Authors:** Jingjing Liu, Yujie Zhu, Yuhao Zhang, Aiying Guo, Mengjiao Li, Jianhua Zhang

**Affiliations:** 1 Shanghai Key Laboratory of Chips and Systems for Intelligent Connected Vehicle, School of Microelectronics, Shanghai University, Shanghai 200444, China; jjliu@shu.edu.cn (J.L.); zhuyujie@shu.edu.cn (Y.Z.); gayshh@shu.edu.cn (A.G.); 2 State Key Laboratory of Integrated Chips and Systems, Fudan University, Shanghai 201203, China; 21112020123@m.fudan.edu.cn

**Keywords:** cross-modal image fusion, multi-sensor, learned low-rank representation, convolutional sparse coding, multi-level optimization

## Abstract

With the development of cross-modal image fusion in multi-sensor systems, current fusion technologies have made significant progress in feature extraction, facilitating more effective image analysis. However, insufficient fusion information may degrade the correlation between the source and fused images, often resulting in the omission of critical features from the original modalities. Therefore, in order to preserve as much information as possible, especially for the complete extraction of effective feature information in source images, this paper proposes a new cross-modal image fusion method based on low-rank representation and convolutional sparse learning named LRCFuse. Firstly, the learned low-rank representation (LLRR) blocks are employed to perform dimensionality reduction on the source images while simultaneously extracting their low-rank and sparse feature components. Nevertheless, considering that the low-rank representation has insufficient modeling ability for different modal images, we introduce common feature preservation module (CFPM) blocks based on convolutional sparse coding. By leveraging the CFPM module, LRCFuse recovers common features from both source images to mitigate the loss caused by the imperfect assumptions of low-rank representation. Based on this, a multi-level optimization strategy incorporating pixel loss, shallow-level loss, mid-level loss, deep-level loss, and sobel loss is proposed to hierarchically learn and refine diverse image features. Quantitative and qualitative evaluations are conducted across various datasets, revealing that LRCFuse can effectively detect targets infrared salient targets, preserve additional details in visible images, and achieve better fusion results for subsequent downstream tasks.

## 1. Introduction

Image fusion is a technique that integrates information from multi-modal sensors to generate a more comprehensive feature representation. Its core objective is to overcome the limitations of a single sensor in terms of accuracy, resolution, dynamic range, and noise resistance, thereby providing more reliable input for high-level visual analysis and decision making. Fused images can typically retain the complementary features of multiple sources of data, and thus play an irreplaceable role in various fields such as military monitoring [1], medical imaging [2], and environmental perception [3].

The current fusion methods can be classified into two categories: traditional methods [4,5,6] and deep learning methods [7,8,9]. Traditional methods, such sparse representation (SR), can extract sparse feature representations from the original data, which can reveal the structure and patterns of the data and have better mathematical interpretability. Dalal et al. [10] and Wright et al. [11] laid the foundation for feature representation learning by formulating images as sparse linear combinations and minimizing reconstruction error. Yao et al. [12] further proposed a joint sparse representation (JSR) model using a shared dictionary to achieve the collaborative fusion of multi-source images. Although SR can effectively capture local details, it has obvious limitations in modeling the global structure. To solve this problem, Candes et al. [13] introduced the robust principal component analysis (RPCA) method, which decomposes the data into low-rank components and sparse components, thereby capturing the global structure and local anomalies. Li et al. [14] adopted low-rank representation (LRR) instead of sparse representation (SR) and represented the data in the form of a low-rank matrix, which is suitable for noise data removal. To address the limitations of image reconstruction (SR) in capturing the global structure, Wu et al. [15] introduced a new representation learning technique using latent LRR (LatLRR), which can extract global and local structure information from the source images. Zhang et al. [16] proposed tensor low-rank representation (TLRR), which imposes low-rank constraints in a higher-order tensor domain to maintain cross-modal consistency. Although these methods have clear mathematical models, they usually rely on artificial prior knowledge and iterative optimization. Their computational complexity is high and their generalization ability for complex scenarios is also limited.

In contrast, the deep learning method, with its powerful end-to-end feature learning capability, significantly improves the performance of image fusion [17]. Li et al. [18] utilized dense connection architecture (DenseFuse) to enhance feature flow. Zhang et al. [19] provide a universal and efficient convolutional neural network (CNN) fusion framework (IFCNN). Its success essentially lies in the replacement of model-driven approaches by data-driven approaches, integrating the processes of feature extraction, activation and fusion into an efficient forward propagation network through extensive supervised or unsupervised training. Zhou et al. [20] used an M-SSIM- and TV-based hybrid loss to adaptively fuse infrared thermal features with visible textural, eliminating the need for manual fusion rules. Li et al. [21] combined a dual-stream feature interaction architecture with a color transformation network to preserve thermal target information while enhancing background texture and color fidelity. However, CNNs are inherently limited by their local receptive fields, making it difficult to effectively model long-range dependencies and global contextual information. To overcome this limitation, generative adversarial networks (GANs) has been introduced into fusion tasks. Ma et al. [22] pioneered the integration of adversarial training of FusionGAN into fusion tasks, generating high-quality fusion results through the interaction between the generator and the discriminator. Subsequently, Xu et al. [23] proposed a unified unsupervised framework (U2Fusion) that adaptively retains the salient information from the source images. Li et al. [24] utilized the adversarial learning between the generator and the discriminator to enhance the perceptual realism of the fused images. Meanwhile, the Transformer architecture and its core self-attention mechanism have also been introduced into this field. Ma et al. [25] presented the Swin Transformer image fusion network (Swin Fusion), which explicitly models global relationships through self-attention mechanisms, significantly improving contextual awareness, albeit at the cost of substantially increased computational complexity and parameter volume. To balance global modeling and multi-scale feature extraction, Shen et al. [26] designed a multi-scale cross-modal attention mechanism to enhance the extraction of significantly complementary features between different modalities. Zhang et al. [27] proposed a dual-branch frequency–spatial joint perception cross-modality network, specifically for improving the fusion quality of significant regions. Cheng et al. [28] integrated a joint low-light enhancement and image fusion network with channel attention to enhance fusion performance under low-light conditions. Li et al. [29] proposed a fusion method based on Knowledge Distillation and the Kansformer architecture, which achieves efficient multi-modal feature modeling under a dynamic sparse attention mechanism through a “Passive–Active” distillation learning scheme. Although these methods have significantly improved performance, they have poor mathematical interpretability and face bottlenecks in terms of credibility and reliability in critical applications.

To address the dual challenges of insufficient interpretability in deep learning and the limited generalizability of traditional methods, researchers have begun to explore a hybrid paradigm that combines traditional sparse priors with deep learning architectures, aiming to maintain the powerful feature learning capabilities of deep networks while preserving the mathematical transparency of traditional models. Li et al. [30] proposed RFN-Nest, which optimizes the information flow through residual fusion networks and nested connection structures. This method introduces dense skip connections and nested residual paths in the encoder–decoder architecture, effectively enhancing the integration and transmission of multi-scale features, significantly improving the detail restoration ability, and simultaneously alleviating the gradient vanishing problem in deep networks. On this basis, Li et al. [31] presented a lightweight dual-branch hybrid architecture. This architecture utilizes CNN and Transformer modules in parallel to extract local features and global dependencies respectively, and introduces an adaptive gating mechanism to achieve feature selection and fusion. It significantly reduces computational complexity while maintaining excellent fusion performance. On the other hand, in order to better fuse the complementary information in infrared and visible light images, Wang et al.  [32] proposed an adaptive interactive Transformer and can effectively highlight infrared targets (AITFuse), while retaining the visible light background texture through cross-modal feature interaction and adaptive weight learning. Liu et al. [33] developed MATCNN technique, which combines multi-scale adaptive transform convolution and efficient attention mechanisms, embedding deformable convolution and spatial attention in the convolution structure to achieve cross-scale feature fusion and robust adaptation to geometric changes with lower parameter quantities. These hybrid methods not only enhance performance but also provide new avenues for understanding the decision-making mechanism of deep networks by introducing mathematical constraints from traditional priors.

Despite the success of these hybrid approaches in balancing interpretability and performance, the rapid evolution of deep learning has witnessed the emergence of entirely new architectural paradigms that operate through fundamentally different mechanisms. In recent years, Diffusion models and Mamba architectures have achieved remarkable progress in computer vision. Diffusion models generate high-quality images through iterative denoising processes, demonstrating exceptional performance in generative tasks such as super-resolution and image inpainting. Ma et al. [34] introduced the Diffusion model to the infrared and visible light fusion task, by combining the fusion prior with the iterative denoising process through the conditional diffusion framework, and using the reverse process to generate high-quality fused images, this effectively alleviated the instability problem in GAN training and enhances the ability to retain details. Mamba architectures, based on selective state space modeling, efficiently capture long-range dependencies with linear complexity, showing superior performance in classification and segmentation tasks. Xie et al. [35] proposed a dynamic feature enhancement network based on Mamba, which utilized the linear complexity selective scanning mechanism of the state space model to capture long-range dependencies, combined reversible neural network blocks to achieve lossless information transmission, and achieved efficient global modeling and local detail preservation in infrared–visible light fusion and medical image fusion tasks. However, these methods primarily focus on data-driven implicit feature learning, making it difficult to explicitly model the low-rank structure and sparse characteristics of multi-modal images. Consequently, they lack interpretability and deterministic guarantees for infrared and visible image fusion tasks. While these emerging paradigms push the boundaries of representational capacity, they also reveal a critical gap in explicit structural priors that could bridge interpretability and performance. This limitation motivates a reconsideration of classical model-driven approaches that enforce sparsity and low-rank constraints through principled mathematical formulations.

Convolutional sparse coding (CSC), as the theoretical foundation of CNNs, formulates image generation as a convolution of filter dictionaries and sparse feature maps, where the sparsity constraint can be regarded as a form of hard attention. In contrast, self-attention mechanisms can be interpreted as a soft, data-driven sparsification process. This theoretical link offers new perspectives for combining model interpretability with data-driven representational power. In recent years, researchers have attempted to integrate traditional priors with deep learning. For instance, Huang et al. [36] designed a two-stage fusion strategy based on CSC, which combines feature transfer and supplementation to enhance modal alignment. Yang et al. [37] proposed the LatLRR-CNN framework combining low-rank representation and CNNs, which combines feature transfer and supplementation to enhance modal alignment. By combining low-rank representation with CNNs, the trade-off between fusion quality and model complexity is effectively achieved. On this basis, Li et al. [38] further designed LRRNet, which combines the traditional low-rank representation theory with deep learning models. By introducing a low-rank regularization term to constrain the feature learning process, it can maintain the consistency of image structure while improving the detail retention ability and enhancing the visual naturalness and structural integrity of the fusion results. Yu et al. [39] incorporated CSC and attention mechanisms to enhance multi-modal feature integration. Cao et al. [40] proposed a cross-domain perception attention mechanism. By constructing a unified cross-domain feature representation and projecting it into the spatial and spectral subspaces, it effectively captures the intrinsic dependency relationship between space and spectrum. On this basis, a frequency-domain perception module is further introduced to fully utilize the low-level statistical features and high-level semantic features, thereby enhancing the comprehensive ability of structural and semantic representations. Based on this, Liu et al. [41] employed plug-and-play (PnP) low-rank priors for efficient dictionary learning, which learns effective feature maps through plug-and-play low-rank prior learning, and requires all sparse feature maps to be highly correlated at the global dimension, which precisely mimics the global context perception ability pursued by the self-attention mechanism. These works show that a hybrid approach, leveraging both interpretable traditional priors and deep learning, effectively constructs image fusion results of high performance.

**Figure 1:**
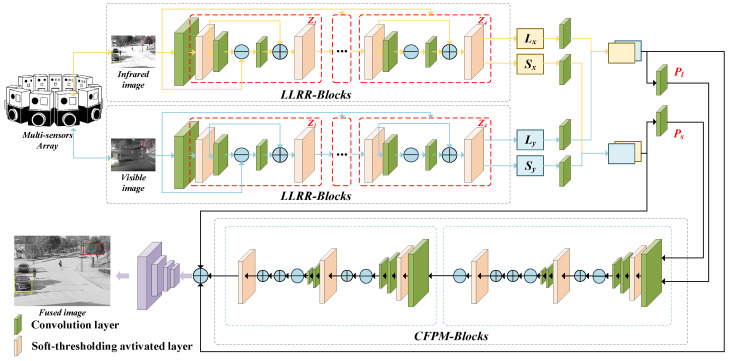
Overview framework of the proposed LRCFuse method. The red frame denotes an LLRR-Block, and the blue frame denotes a single CPFM-Block. *L* and *S* denote the low-rank and sparse components extracted from the source images, respectively. $P_{l}$ represents the fused low-rank feature part, and $P_{s}$ represents the fused sparse feature part. *Z* denotes the output of the LLRR-Block module.

Inspired by this, we propose an end-to-end image fusion framework base on low-rank representation and CSC named LRCFuse, whose structure is illustrated in Figure 1. The method first employs a low-rank representation learning module to extract low-rank and sparse components ($L_{x}$, $L_{y}$, $S_{x}$, and $S_{y}$) from the input images. *Z* represents the output of the LLRR-Block, while $P_{l}$ and $P_{s}$ represent the low-rank and sparse feature parts extracted from the two source images. Simultaneously, a common feature preservation module (CFPM) is introduced to extract cross-modal common features $C_{f}$. Finally, the three types of features are aggregated to reconstruct the fused image. Furthermore, a multi-level optimization strategy is designed to comprehensively enhance fusion quality, ensuring that the output closely aligns with the source images in both structure and details.

The main innovations of this paper can be summarized as follows:

- **Novel Fusion Method.** This paper introduces a new cross-modal image fusion method, named LRCFuse, utilizing common feature preservation module (CFPM) to retain common features, and it employs learned low-rank representation (LLRR) to extract useful low-rank and sparse features, which is highly memory efficient for deployment.
- **Multi-level Optimization.** This work introduces a new multi-level optimization containing five loss terms, including pixel loss, shallow, middle, deep loss, and sobel loss, to improve model performance at multiple scales through image details, feature levels, and edge information.
- **Applications.** Experiments are conducted on accessible visible and infrared image datasets and compared with the latest approaches, show that the proposed LRCFuse achieves better information retention and image contrast.

The rest of this paper is organized as follows. Section 2 provides a brief overview of relevant works related to the topic of this paper. A detailed illustration of LRCFuse is presented in Section 3. The experimental results and analysis of LRCFuse are presented in Section 4. Section 5 summarizes the work of the paper and provides future research directions.

## 2. Related Work

Given an input data matrix $X\in R^{n_{1}\times n_{2}}$, where $n_{1}$ and $n_{2}$ represent the dimensions of each pattern, the objective of RPCA is to decompose *X* into a low-rank matrix *L* and a sparse matrix *S* by solving a convex optimization problem that minimizes a weighted combination of the nuclear norm of *L* and the $l_{1}$-norm of *S*.

(1) $$\begin{array}{cc} \min_{L,S} & {∥L∥}_{*}+\lambda {∥S∥}_{1}\\ s.t. & X=L+S, \end{array}$$

where ${∥\cdot ∥}_{*}$ and ${∥\cdot ∥}_{1}$ represent the nuclear norm and the $l_{1}$-norm, facilitating the low-rank and sparse recovery, respectively. While the minimization of these norms enables RPCA to successfully decompose the data into a low-rank background and a sparse anomaly component, a well-known limitation of this framework is its inability to efficiently model local spatial dependencies.

To address these limitations, the low-rank representation (LRR) model seeks to represent the data within a unified low-rank subspace. This framework retains the strength of global low-rank modeling inherent to RPCA but demonstrates superior efficacy in processing high-dimensional data and revealing latent structures, which are particularly vital in applications like image fusion. This can be formally expressed by the following optimization problem:

(2) $$\begin{array}{cc} \min_{Z,E} & {∥Z∥}_{*}+\lambda {∥E∥}_{1}\\ s.t. & X=D*Z+E,Z=L+S, \end{array}$$

where ∗ denotes the convolution operator, *D* represents the dictionary matrix, and *Z* is globally constrained by the nuclear norm ${∥Z∥}_{*}$, and is decomposed into the low-rank matrix *L* and the sparse matrix *S*. Under this decomposition, the problem Equation (2) is transformed into

(3) $$\begin{array}{cc} \min_{L,S,E} & {∥L∥}_{*}+\lambda_{1}{∥S∥}_{F}+\lambda_{2}{∥E∥}_{1}\\ s.t. & X=D*(L+S)+E, \end{array}$$

where ${∥\cdot ∥}_{F}$ denotes the Frobenius norm. Although LRR demonstrates competent performance in capturing low-rank and sparse components, they operate inherently within the matrix framework, thereby failing to fully preserve the high-order structural and multimodal correlations present in real-world data. To address this limitation, Zhou et al. [42] proposed the tensor LRR (TLRR) model, which extends the low-rank constraint from the matrix to the tensor domain, where ${∥L∥}_{*}$ denotes the nuclear norm based on the tensor singular value decomposition (t-SVD). However, the tensor nuclear norm based on t-SVD usually suffers from high computational cost and complex iterative optimization.

To address this issue, Yang et al. [37] proposed an infrared and visible image fusion method, termed LatLRR-CNN, which combines latent low-rank representation (LatLRR) with convolutional neural networks (CNN). In this method, LatLRR is employed to decompose the source images into low-rank parts and salient parts, as shown in Equation (4), which are then fused separately by CNNs. By introducing the self-representation term XZ and the latent low-rank component LX, LatLRR avoids heavy tensor decomposition while still effectively capturing the low-rank structure of the data, leading to the formulation in Equation (4).

(4) $$\begin{array}{cc} \min_{Z,L,E} & {∥Z∥}_{*}+{∥L∥}_{*}+\lambda {∥E∥}_{1}\\ s.t. & X=XZ+LX+E, \end{array}$$

However, the LatLRR model in Equation (4) still relies on the conventional nuclear norm to enforce low-rankness, which limits its efficiency and representation capability. To overcome this issue, Liu et al. [41] employed the plug-and-play tensor low-rank approximation (PnP-TLRA) framework in Equation (5). In this framework, the standard nuclear norm is replaced with a plug-and-play (PnP) prior based on deep learning, enabling more efficient and flexible low-rank representation.

(5) $$\begin{array}{cc} \min_{L,S,E} & {∥L∥}_{*}+\lambda_{1}{∥S∥}_{F}+\lambda_{2}{∥E∥}_{1}^{2}\\ s.t. & X=D*L+S+E, \end{array}$$

where the ${∥\cdot ∥}_{*}$ strategy incorporates deep learning-based PnP priors to guide and constrain the low-rank estimation.

Although the PnP-TLRA framework has shown strong performance, it still suffers from several inherent limitations. Specifically, the model heavily relies on global low-rank constraints, which are often inadequate for capturing fine-grained local structures. The optimization process tends to emphasize global correlations at the expense of localized fidelity, potentially leading to oversmoothed outputs.

## 3. The Proposed Method

To overcome the limitations of existing cross-modal fusion approaches, we propose a novel framework named LRCFuse. The architecture of LRCFuse is organized into three parts. First, the LLRR module decomposes the source images into low-rank and sparse components through a decomposition model and its corresponding LLRR-Blocks. Next, the CFPM module, based on convolutional sparse coding, is introduced to recover common features shared across modalities, thereby compensating for the insufficient modeling ability of low-rank representation alone. Finally, a multi-level loss function is designed, incorporating pixel, shallow, middle, deep, and sobel constraints, in order to hierarchically supervise and refine the fused representation. The details of the LLRR, the CFPM, and the loss formulation are described in the following subsections.

### 3.1. LLRR

#### 3.1.1. The Decomposition Model

Benefiting from the strengths of the above three formulations (Equations (1)–(5)) in terms of low-rank modeling, foreground preservation, and computational efficiency, we further propose a multi-dictionary low-rank and sparse decomposition model in Equation (6), where the input *X* is decomposed as $X=D_{1}L+D_{2}S+E$, with *L* representing the low-rank matrix, *S* denoting the sparse matrix, $D_{1}$ and $D_{2}$ denoting the dictionaries for the low-rank and sparse components, and *E* reinterpreted as a noise. This formulation jointly imposes refined constraints on the low-rank term, sparse structure, and noise residuals, while employing independent dictionaries to decouple different components. As a result, it enhances the interpretability and structural modeling capacity of the fused features.

(6) $$\begin{array}{cc} \min_{L,S,E} & {∥L∥}_{*}+\lambda_{1}{∥S∥}_{F,p}+\lambda_{2}{∥E∥}_{F,p}^{2}\\ s.t. & X=D_{1}L+D_{2}S+E, \end{array}$$

Here, $l_{F,p}$ (with 0≤p≤1) denotes a joint sparse norm promoting structured sparsity. Unlike the standard $l_{1}$-norm, which induces element-wise sparsity, the $l_{F,p}$-norm encourages group-wise sparsity by combining the $l_{2}$-norm of rows within the matrix. This construction drives entire rows to zero when their collective energy is small, making it particularly effective for tasks such as joint feature selection or image fusion, while preserving the internal relationships among coefficients in non-zero rows, as shown in Equation (7).

(7) $$\begin{array}{cc} \min_{L,S,E} & {∥L∥}_{*}+\lambda_{1}{∥S∥}_{2,p}+\lambda_{2}{∥E∥}_{2,p}\\ s.t. & X=D_{1}L+D_{2}S+E,0\leq p\leq 1, \end{array}$$

We incorporate the PnP prior and adopt the DDLCN [43] architecture to learn two discriminative dictionaries, $D_{1}$ and $D_{2}$. These dictionaries are used to project the low-rank structure *L* and the sparse structure *S* into a shared representation space. In this model, the term $\left(D_{1}L+D_{2}S\right)$ captures the essential image content, encompassing both global structures and latent mappings. To regularize the noise term *E*, we employ the generalized $l_{2,p}$ norm, which promotes structured sparsity and offers flexibly in adapting to deiverse foreground patterns. This idea is motivated by the weighted schatten minimization method proposed by Xu et al. [44], which has demonstrated exceptional performance in image denoising. In their study, when p=1, the model is able to better approximate the original low-rank assumption and effectively remove noise. Motivated by LRR-based formulations, Equation (7) can be rewritten into a unified optimization form as

(8) $$\begin{array}{cc} \min_{D_{1},D_{2},L,S,E} & {∥X-D_{1}L-D_{2}S-E∥}_{F}^{2}+\lambda_{1}{∥L∥}_{*}+\lambda_{2}{∥S∥}_{2,1}\\ & +\;\lambda_{3}{∥E∥}_{2,1}+\lambda_{4}(∥D_{1}{∥}_{F}^{2}+∥D_{2}{∥}_{F}^{2}), \end{array}$$

To quickly optimize Equation (8), the optimization methods for the nuclear norm and Frobenius norm [45] are employed here to avoid time-consuming matrix decomposition calculations. The mapping formula is given as

(9) $$\begin{array}{cc} {∥L∥}_{*} & =\min_{A,B}\frac{1}{2}{∥A∥}_{F}^{2}+\frac{1}{2}{∥B∥}_{F}^{2}\\ s.t. & AB=L, \end{array}$$

By integrating Equations (8) and (9), the issue can be rephrased as follows:

(10) $$\begin{array}{cc} \min_{D_{1},D_{2},A,B,S,E} & {∥X-D_{1}AB-D_{2}S-E∥}_{F}^{2}+\lambda_{1}{∥S∥}_{2,1}+\frac{\lambda_{2}}{2}\left({∥A∥}_{F}^{2}+{∥B∥}_{F}^{2}\right)\\ & +\;\lambda_{3}{∥E∥}_{2,1}+\lambda_{4}\left(∥D_{1}{∥}_{F}^{2}+{∥D_{2}∥}_{F}^{2}\right), \end{array}$$

Finally, the model can be stated in a general concise form as

(11) $$\begin{array}{c} \min_{D,Z,E}\frac{1}{2}{∥X-DZ-E∥}_{F}^{2}+\lambda_{3}{∥E∥}_{2,1}+\lambda_{4}{∥D∥}_{F}^{2}+\psi \left(Z\right), \end{array}$$

where $D=(D_{1},D_{2})$, Z=(AB;S), and $\psi \left(Z\right)=\lambda_{1}{∥S∥}_{2,1}+\frac{\lambda_{2}}{2}({∥A∥}_{F}^{2}+{∥B∥}_{F}^{2})$ denote the regularization term combining nuclear norm approximation and sparse error. Then, in order to solve Equation (11), we propose the optimization algorithm for LRCFuse method (see Algorithm 1).

In Algorithm 1, $W_{e}$ denotes the encoding matrix, which projects the input (X−E) into the coefficient space. *U* denotes the constant term obtained by projecting the input (X−E) through the encoding matrix $W_{e}$, which serves as the driving signal for the iterative update. In the formula $H={\hat{\lambda }}_{4}I-\mu D^{⊤}D$, *V* denotes the identity matrix, and *H* represents the linear recurrence/contraction matrix to enhance stability. μ is the step size, set as $\mu =1/{∥D∥}_{2}^{2}$. The $P_{l}=D_{1}L$ and $P_{s}=D_{2}S$ are then reconstructed with the learned dictionary $D_{1}$ and $D_{2}$.
**Algorithm 1** Optimization algorithm for LRCFuse with $D_{1}$ and $D_{2}$.**Input**: $X,D_{1},D_{2},E,\lambda_{1},\lambda_{2},\lambda_{3},\lambda_{4}.$**Output**: $P_{l},P_{s}.$**Define 1**: $D=\left(D_{1},D_{2}\right),{\hat{\lambda }}_{4}=1-\lambda_{4}.$**Define 2**: $H={\hat{\lambda }}_{4}V-\mu D^{T}D,\;W_{e}=\mu D^{T},\;\theta =\mu \left(\begin{array}{c} \lambda_{1}\\ \lambda_{2}\\ \lambda_{3} \end{array}\right).$1:Initialize $U=W_{e}\left(X-E\right),\;Z_{0}=h_{\theta }\left(U\right)$2:**for** t=1 to *T* **do**3:$\;Z_{t}^{\prime }=U+HZ_{t-1}$4:$\;Z_{t}=h_{\theta }\left(Z_{t}^{\prime }\right)$5:**end for**6:Let $Z=Z_{T}$ and split Z=(AB,S), where L=AB is the low-rank matrix and *S* is the sparse matrix.7:Output $P_{l}=D\left(AB\right)$ and $P_{s}=DS$.

Consequently, the iterative optimization in Algorithm 1 can be reformulated as

(12) $$\begin{array}{cc} Z_{t} & =h_{\theta }\left(B+HZ_{t-1}\right)\\ \; & =h_{\theta }\left(W_{e}\left(X-E\right)+\hat{\lambda_{4}}Z_{t-1}-W_{e}DZ_{t-1}\right)\\ \; & =h_{\theta }\left(W_{e}\left(X-E-DZ_{t-1}\right)+\hat{\lambda_{4}}Z_{t-1}\right)\\ \; & =h_{\theta }\left(C_{2}*\left(X-E-C_{1}*Z_{t-1}\right)+\hat{\lambda_{4}}Z_{t-1}\right), \end{array}$$

where ∗ indicates the convolution operation, $C_{1}$ and $C_{2}$ are learnable convolutional layers, and $h_{\theta }$ refers to the soft thresholding (shrinkage) function [46]. Here $h_{\theta }$ is an activation function, which is defined as follows:

(13) $$\begin{array}{c} h_{\theta }\left(X\right)=\mathrm{sign}\left(X\right)\cdot \max\left(|X|-\theta ,0\right), \end{array}$$

where sign(·) indicates the sign function. The output of a stack of LLRR blocks produces the final features *Z* (steps in LISTA [47]). The low-rank feature $\left(P_{l}=C_{l}*L\right)$ and the sparse feature $\left(P_{s}=C_{s}*S\right)$ can be computed with the proper convolutional layers ($C_{l}$ and $C_{s}$). Here, convolutional layers can be regarded as a practical implementation of dictionaries, namely convolutional dictionaries. Therefore, $D_{1},\;D_{2}$ and $C_{l},\;C_{s}$ are mathematically equivalent, with the former being more common in theoretical formulations and the latter more suitable for describing network implementations.

Next, the common features *C* will be obtained by solving the following optimization formula:

(14) $$\begin{array}{cc} \underset{\left\{C,L,S\right\}}{\mathrm{Argmin}}\; & \frac{1}{2}{∥X-\sum_{k}\left(C_{3}*P_{c}+C_{4}*P_{l}\right)∥}_{2}^{2}\\ + & \frac{1}{2}{∥Y-\sum_{k}\left(C_{5}*P_{c}+C_{6}*P_{s}\right)∥}_{2}^{2}\\ + & \lambda \sum_{k}\left({∥P_{c}∥}_{1}+{∥P_{l}∥}_{1}+{∥P_{s}∥}_{1}\right), \end{array}$$

where $C_{3},\;C_{4},\;C_{5}$, and $C_{6}$ represent the four convolution layers. *X* and *Y* represent the visible light and infrared images respectively. Equation (14) is solved by updating each variable alternately while fixing the other variables, and the iterative solution process can be referred to in the literature [9]. Then, the proposed common feature part $P_{c}$ can be obtained. Finally, the $P_{c}$, $P_{l}$ and $P_{s}$ obtained through Equation (14) are summed pixel by pixel and averaged to obtain the final fused feature values. Then, three layers of deconvolution are applied to obtain the final fused image.

#### 3.1.2. LLRR-Blocks

**Figure 2:**
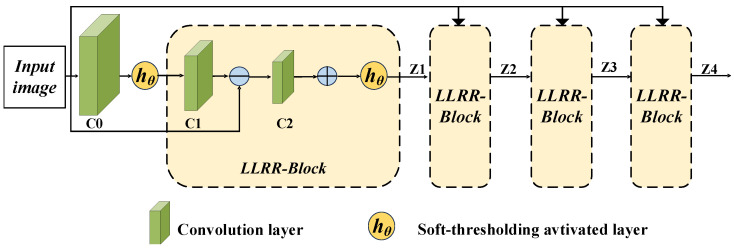
The architecture of LLRR-Blocks. This module consists of multiple cascaded LLRR-Blocks, each integrating convolutional layers, local residual connections, and a soft thresholding activation function ($h_{\theta }$).

After applying the LLRR decomposition model to a single-channel image to extract its low-rank and sparse features, multiple LLRR layers are cascaded to construct deep representations. The structure of the LLRR-Block is shown in Figure 2, where the output feature *Z* is obtained via optimization using the LISTA algorithm. The core of LISTA is the soft thresholding operation, which updates the low-rank and sparse coefficients in each iteration.

In the LRCFuse framework, four LLRR blocks are employed to decompose the source images (infrared image $I_{ir}$ and visible image $I_{vi}$, as shown in Figure 1). Specifically, $L_{x}$ and $S_{x}$ denote the low-rank and sparse coefficients of the visible image, respectively, while $L_{y}$ and $S_{y}$ represent the corresponding coefficients for the infrared image. Each LLRR block progressively extracts residual information from the source features, ensuring that subsequent modules capture characteristics not identified in previous layers, thereby retaining more details and critical information step by step.

The separation of sparse features is primarily achieved through the soft thresholding function $h_{\theta }$, which operates on the feature map in each iteration of LISTA. This function thresholds the features by setting values below θ to zero, while shrinking significant features, thereby suppressing noise and non-significant components and enhancing the sparsity of the features.

After obtaining the low-rank and sparse $L_{x}$, $S_{x}$, $L_{y}$, and $S_{y}$, four convolutional layers $C_{1}$, $C_{2}$, $C_{3}$, and $C_{4}$ are used to further extract their respective feature representations. These are then fused through concatenation and two additional convolutional layers, ultimately yielding the fused low-rank feature $L_{f}$ and sparse feature $S_{f}$.

### 3.2. CFPM-Blocks

In the above work, the LLRR-Blocks first decompose the features into a low-rank component $L_{f}$ and a sparse component $S_{f}$. Subsequently, the CFPM-Blocks formulate the set of common feature coefficients $C_{f}=\left\{C_{f,k}\right\}$ as the optimization variables and conduct a joint regularized reconstruction of both modalities under a sparsity prior, as specified in Equation (15).

(15) $$\begin{array}{cc} C_{f}= & \arg\min_{C_{f}}\frac{1}{2}{∥\;X-\sum_{k}\;\left(C_{3,k}*C_{f,k}+C_{4,k}*L_{f,k}\right)\;∥}_{2}^{2}\\ \; & +\frac{1}{2}{∥\;Y-\sum_{k}\;\left(C_{5,k}*C_{f,k}+C_{6,k}*S_{f,k}\right)\;∥}_{2}^{2}\;+\;\lambda \sum_{k}{∥C_{f,k}∥}_{1}, \end{array}$$

where the convolution kernels $C_{3,k}$ and $C_{5,k}$ are used to capture cross-modal structural patterns, while $C_{4,k}$ and $C_{6,k}$ are convolved with the known low-rank $L_{f}$ and sparsity $S_{f}$, respectively, to incorporate modality-specific decomposable elements. In addition, $C_{f}={\left\{C_{f,k}\right\}}_{k=1}^{K}$ represents a set of shared feature coefficient maps, where each convolutional kernel $C_{3,k},C_{4,k},C_{5,k},$ and $C_{6,k}$ corresponds to the *k*-th coefficient and is convolved respectively with $C_{f,k}$, $L_{f,k}$, $C_{f,k}$, and $S_{f,k}$, during the reconstruction of the two modalities. Here, $L_{f,k}$ and $S_{f,k}$ represent the $L_{f}$ and $S_{f}$ aligned with the channel or sub-band associated with the *k*-th kernel. The parameter λ controls the strength of the $l_{1}$ regularization term.

**Figure 3:**
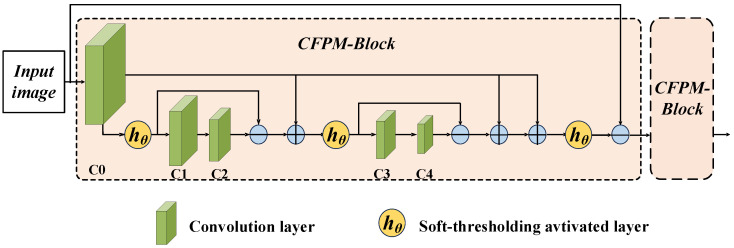
The architecture of CFPM-Blocks. The CFPM-Blocks build upon convolutional layers and soft thresholding activation functions ($h_{\theta }$). It integrates dense-like internal skip connections, which propagate early-stage features to multiple subsequent stages, together with local residual connections.

Given that $L_{f}$ and $S_{f}$ have been derived from the LLRR-Block module, the CFPM module first treats them as known priors to strip away the explainable components of each modality, resulting in two residuals signals for the subsequent shared convolutional fitting. These residuals are then jointly represented under convolutional kernel sets $\left\{C_{3,k}\right\}$ and $\left\{C_{5,k}\right\}$ via CSC, and the shared coefficients $C_{f}$ are estimated with an additional $l_{1}$ sparsity regularization constraint. This process directly yields $C_{f}$ by minimizing the objective function. This strategy explicitly decouples modality-specific low-rank and sparse information from cross-modal shared representations by first separating the unique components and then encoding the residuals with shared convolutional sparse coding, thereby enhancing the interpretability and robustness of the common features. To ensure the reproducibility of the proposed method and clarify the alternate updating process of the CFPM-Blocks, the detailed step-by-step optimization procedure is summarized in Figure 3 and Algorithm 2.
**Algorithm 2** Optimization process for CFPM-Blocks  1:**Input:**$L_{f}$, $S_{f}$, *X* and *Y*.  2:**Output:**$C_{f}$.  3:**Initialize:**$C_{f}^{\left(0\right)}=0$, learning rate η, regularization parameter λ.  4:**Step 1: Residual Extraction (Pre-processing)**  5:    $R_{X}=X-\sum_{k}\left(C_{4,k}*L_{f,k}\right)$ {Extract residuals from modality X}  6:    $R_{Y}=Y-\sum_{k}\left(C_{6,k}*S_{f,k}\right)$ {Extract residuals from modality Y}  7:**Step 2: Iterative Optimization (Unfolded into*****T*****Stages)**  8:**for**t=1**to***T***do**  9:    {Gradient Descent Step for Fidelity Terms in Equation (15)}10:    $\nabla^{\left(t\right)}=\frac{\partial }{\partial C_{f}}\left(\frac{1}{2}{∥R_{X}-\sum_{k}\left(C_{3,k}*C_{f,k}\right)∥}_{2}^{2}+\frac{1}{2}{∥R_{Y}-\sum_{k}\left(C_{5,k}*C_{f,k}\right)∥}_{2}^{2}\right)$11:    $Z^{\left(t\right)}=C_{f}^{\left(t-1\right)}-\eta \nabla^{\left(t\right)}$ {Implemented by Convolution Layers $C_{1},C_{2}$}12:    {Proximal Mapping for $l_{1}$ Sparsity Regularization}13:    $C_{f}^{\left(t\right)}=\mathrm{soft}\text{\_}\mathrm{thresholding}(Z^{\left(t\right)},\lambda )$ {Implemented by $h_{\theta }$ Layer}14:**end for**15:$C_{f}=C_{f}^{\left(T\right)}$ {Assign the final iterated value to $C_{f}$}16:**return**$C_{f}$

### 3.3. The Loss Functions of LRCFuse

**Figure 4:**
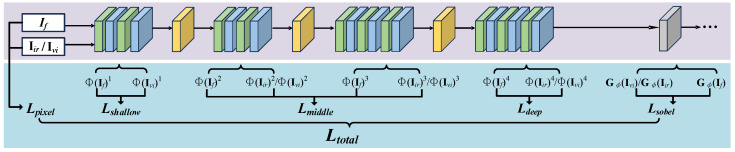
The fused image and the original images are input to the pre-trained network to compute the loss function. It illustrates the process by which the fused image and the original images are fed into a pre-trained network, and the loss function for evaluating fusion performance is computed via the network’s shallow, middle, and deep feature extraction and transformation modules.

To ensure that the fused image preserves complementary information from both infrared and visible source images, the generated fused image $I_{f}$, the visible image $I_{vi}$, and the infrared image $I_{ir}$ are fed into a pre-trained VGG-16 [48] network. By extracting feature representations $\Phi {\left(\cdot \right)}^{k}$ at different convolutional layers, we obtain low-level texture and edge information, mid-level semantic structures, and high-level abstract representations. Based on these hierarchical features, the corresponding loss functions are constructed to guide the training process, ensuring that the fused image remains consistent with the source images at the pixel, structural, and semantic levels. The loss function architecture is shown in Figure 4. The definition of the total loss function $L_{total}$ is as follows:

(16) $$\begin{array}{cc} L_{total}= & \gamma_{1}L_{pixel}+\gamma_{2}L_{shallow}+L_{middle}+\gamma_{4}L_{deep}+\gamma_{5}L_{sobel}, \end{array}$$

where $\gamma_{1}$, $\gamma_{2}$, $\gamma_{4}$, and $\gamma_{5}$ represent the weight of the loss function for each part. For the description of the losses for each part, it is as follows.

- $L_{pixel}$ measures the per-pixel difference between the fused and visible images, encouraging the fused image to remain close to the source and preserve visible details.
  (17) $$\begin{array}{c} L_{pixel}={∥I_{f}-I_{vi}∥}_{F}^{2}, \end{array}$$
  where $I_{f}$ represents the fused image and $I_{vi}$ represents the visible image.
- $L_{shallow}$ uses low-level features to enforce local structural similarity between the fused and visible images, such as edges and textures.
  (18) $$\begin{array}{c} L_{shallow}=||\Phi {\left(I_{f}\right)}^{1}-\Phi {\left(I_{vi}\right)}^{1}{\left|\right|}_{F}^{2}, \end{array}$$
  where Φ(·) represents the features that the loss network extracts from (·), and ${\Phi (\cdot )}^{1}$ represents the output of the first convolution block.
- $L_{middle}$ guides the fusion process to retain critical infrared information and enhance the semantic content of the fused image.
  (19) $$\begin{array}{cc} L_{\mathrm{middle}\;}= & \sum_{k=2}^{K}\beta^{k}{∥\Phi {\left(I_{f}\right)}^{k}-\left[w_{ir}\Phi {\left(I_{ir}\right)}^{k}+w_{vi}\Phi {\left(I_{vi}\right)}^{k}\right]∥}_{F}^{2}, \end{array}$$
  where *K* represents the number of convolutional blocks, and $\beta^{k}$ represents the weight of each item. The balancing parameters $\omega_{ir}$ and $\omega_{vi}$ regulate the trade-off between features in visible and infrared images. In particular, $\omega_{vi}$ ought to be substantially less than $\omega_{ir}$.
- $L_{deep}$ constrains the fused image by matching gram-based correlations [49], thereby enforcing high-level semantic consistency with the infrared source.
  (20) $$\begin{array}{c} L_{\mathrm{deep}\;}={∥\mathrm{Gram}\left(\Phi {\left(I_{f}\right)}^{4}\right)-\mathrm{Gram}\left(\Phi {\left(I_{ir}\right)}^{4}\right)∥}_{F}^{2}, \end{array}$$
  where Gram(·) represents a gram matrix, which is a covariance matrix with no whitening operations.
- $L_{sobel}$ drives the fused image to retain sharper infrared edges and finer structural details.
  (21) $$L_{sobel}=∥G_{\phi }\left(I_{f}\right)-G_{\phi }\left(I_{vi}\right){∥}_{F}^{2}+∥G_{\phi }\left(I_{f}\right)-G_{\phi }\left(I_{ir}\right){∥}_{F}^{2},$$
  Here $G_{\phi }\left(\cdot \right)$ denotes the gradient feature map obtained by the sobel operator, defined as
  (22) $$G_{\phi }\left(I_{f}\right)=\sqrt{{\left(G_{x}\left(I_{vi}\right)\right)}^{2}+{\left(G_{y}\left(I_{vi}\right)\right)}^{2}}+\sqrt{{\left(G_{x}\left(I_{ir}\right)\right)}^{2}+{\left(G_{y}\left(I_{ir}\right)\right)}^{2}},$$
  where $G_{x}\left(I_{vi}\right)$ and $G_{y}\left(I_{ir}\right)$ denote the horizontal and vertical sobel gradients of fused image $I_{f}$, respectively.

In conclusion, the fused image generated by LRCFuse can more comprehensively retain the detailed information in the visible light image. At the same time, by introducing a composite loss function composed of five components and its carefully configured weights, it effectively enhances the saliency of infrared target features.

## 4. Experiments and Analysis

**Figure 5:**
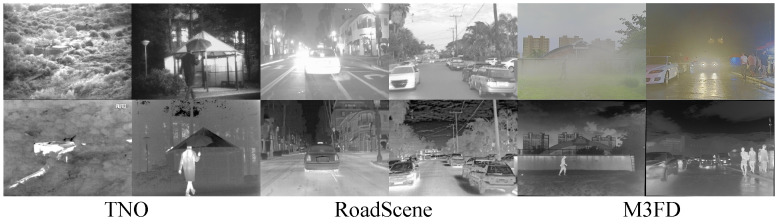
Test datasets. From left to right are TNO, RoadScene and M3FD respectively. The examples of images from these datasets show visible light images (**first row**) and the infrared images (**second row**).

To comprehensively evaluate the performance of LRCFuse, nine state-of-the-art fusion methods are selected for experimental comparison, such as DenseFuse [18], FusionGAN [22], IFCNN [19], U2Fusion [23], RFN-Nest [30], SwinFusion [25], LRRNet [38], AITFuse [32] and MATCNN [33]. Additionally, experiments are conducted on three publicly available datasets: TNO (https://github.com/yanyanchun/TNO_Image_Fusion_Dataset, accessed on 5 March 2026); Roadscene (https://github.com/hanna-xu/RoadScene, accessed on 5 March 2026); and M3FD (https://github.com/JinyuanLiu-CV/TarDAL, accessed on 5 March 2026), as shown in Figure 5, to ensure a robust assessment of the proposed method.

• The TNO dataset, commonly used for visible and infrared image fusion research, contains 60 pairs of cross-modal images captured in military settings. These images cover various environments, including indoor and outdoor scenes, nighttime visual scenarios, challenging lighting conditions, adverse weather conditions, and other diverse scene settings.
• The RoadScene dataset, collected from FLIR videos, is more focused on civilian scenarios, particularly for autonomous driving and intelligent transportation systems. It contains a large number of common road scene elements, such as roads, cars, pedestrians, and other characteristic scenes.
• The M3FD dataset, designed for infrared and visible light image fusion and cross-modal target detection research, is a multispectral dataset. The dataset comprises 15,000 images covering various complex scenes, including six categories of targets such as people, cars, buses, motorcycles, lamps, and trucks.

Furthermore, a comprehensive evaluation of the comparative results is conducted through both qualitative and quantitative assessments. Qualitative evaluation provides intuitive visual comparisons in terms of contrast and structural preservation, yet it is inherently subjective. Quantitative evaluation, on the other hand, employs a set of numerical metrics to characterize fusion performance from multiple perspectives, enabling an objective and multi-faceted analysis. To thoroughly assess the performance of LRCFuse, six evaluation metrics are selected, covering five fundamental aspects of fused image quality: the information-theoretic evaluation metrics En, MI, structural similarity-based evaluation metric SSIM, image feature-based evaluation metric SD, human visual perception-based evaluation metric VIF, and evaluation metric Nabf based on the source image and generated image. For all metrics except Nabf, higher numerical values indicate improved fusion performance.

(1) **En**: Entropy quantifies the information content within an image, with higher values reflecting greater informational richness and uncertainty.

(2) **MI**: Mutual information quantifies the similarity between two images by measuring how much information is shared between them. Higher mutual information indicates a greater degree of similarity.

(3) **SSIM**: The Structural Similarity Index is a widely adopted metric for evaluating image distortion, as it jointly considers luminance, contrast, and structural information. A higher SSIM score indicates improved structural preservation and contrast consistency between the compared images.

(4) **SD**: Standard deviation measures the dispersion of pixel values within an image. A higher standard deviation signifies greater variation in pixel values, making it a useful metric for evaluating image sharpness.

(5) **VIF**: Visual Information Fidelity assesses image quality by considering brightness, contrast, and structural information. A higher Visual Information Fidelity (VIF) score suggests that attributes like brightness and contrast in the fused image are more perceptually aligned with the human visual system.

(6) **Nabf**: Normalized absolute barycenter deviation calculates the average difference between the generated and original images. A lower normalized absolute barycenter deviation indicates that the generated image contains less noise and is closer to the original image.

Finally, the PyTorch 1.13.1 framework is employed to train and test the model on a server running the Ubuntu operating system, equipped with an Intel Core i7-3770k CPU and an NVIDIA GeForce RTX 4090 GPU.

### 4.1. Parameter Settings

#### 4.1.1. Training Details

The proposed model is trained on the MSRS (https://github.com/Linfeng-Tang/MSRS, accessed on 5 March 2026), multispectral dataset. The MSRS dataset is a public benchmark for multi-modal image fusion, consisting of registered pairs of visible and infrared images, and is widely used for training and evaluating cross-modal fusion methods. To generate more training samples, 1444 pairs of infrared and visible image pairs from the MSRS dataset are further processed and divided into 41,703 pairs of patches, each with a resolution of 128 × 128 pixels. From these, 20,000 pairs of IR-VI images are randomly selected for training. The model is trained for 30 epochs with batch size of eight. All training images are converted to grayscale, and the learning rate is set to $1\times 10^{-5}$. Following the work of [38], λ is set to 0.005, $\lambda_{1}$ is set to 0.5, $\lambda_{2}$ to 0.05, and both $\lambda_{3}$ and $\lambda_{4}$ to 0.005.

#### 4.1.2. The Impact of $L_{shallow}$ and $L_{middle}$

**Figure 6:**
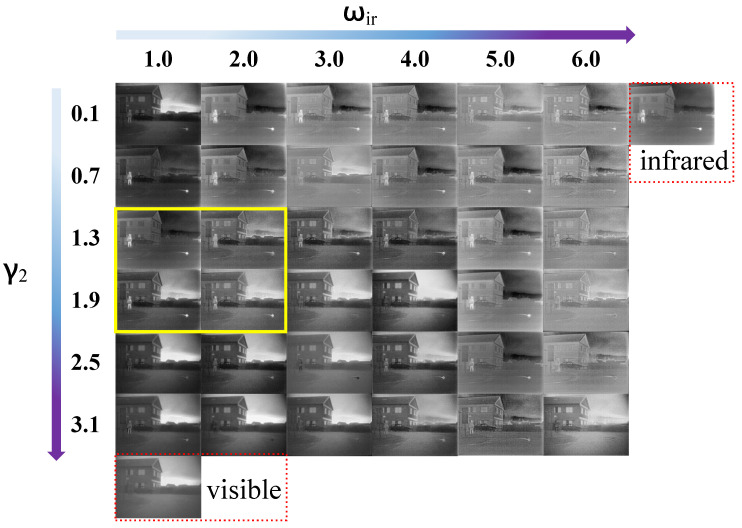
An example of the fusion results obtained with different $\gamma_{2}$ and $\omega_{ir}$. The last row shows a visible image and the right column presents the infrared image. The yellow frames highlight the four groups of results with the best subjective visual effect.

During the training phase, $\beta^{2}$ and $\beta^{3}$ are set to 0.01 and 0.5, respectively, as the value of $L_{middle}$ is significantly larger than the other losses. To maintain more details, $\gamma_{1}$ is set to ten. The $\omega_{vi}$ is set to 0.5 in $L_{middle}$, ensuring it is substantially smaller than $\omega_{ir}$. The number of LLRR-Blocks is fixed at four, with $\gamma_{4}$ is set to 700 in the initial analysis. The influence of parameters ($\gamma_{2}$ in $L_{shallow}$; $\omega_{ir}$ in $L_{middle}$) can be examined under these conditions. Their values are specified as follows: $\gamma_{2}\in \left\{1.5,1.6,1.7,1.8,1.9,2.0\right\}$; $\omega_{ir}\in \left\{1.0,2.0,3.0,4.0,5.0,6.0\right\}$. The fusion results for different value of $\gamma_{2}$ and $\omega_{ir}$ are illustrated in Figure 6.

In the proposed loss function, $\gamma_{2}$ is utilized to control $L_{shallow}$, which represents the shallow information in the image. A larger value of $\gamma_{2}$ leads to more prominent details from the visible light image being fused into the composite image. Meanwhile, the magnitude of $\omega_{ir}$ affects the prominence of infrared features in the fused image. A larger $\omega_{ir}$ results in more prominent infrared features in the fused image. From Figure 6, it is evident that the fused image tends to resemble the visible image more when $\omega_{ir}$ decreases and $\gamma_{2}$ increases, while it resembles the infrared image more when $\omega_{ir}$ increases and $\gamma_{2}$ decreases. Based on Figure 6, a set of combinations can be identified: $\gamma_{2}\in \left\{1.3,1.9\right\}$ and $\omega_{ir}\in \left\{1.0,2.0\right\}$. The average values of the six metrics for different parameter combinations are shown in Table 1.

In Table 1, when $\gamma_{2}$ = 1.9 and $\omega_{ir}$ = 1.0, LRCFuse obtains four optimal values (e.g., En, SD, MI and VIF) and one suboptimal value (SSIM). The four optimal values indicate that LRCFuse retains more detailed information while preserving the salient features of the infrared image. In constrast, the fusion image produced by the other three sets of parameters is not as close to the performance of the original image as those generated by the suboptimal values. Therefore, $\gamma_{2}$ and $\omega_{ir}$ are set to 1.9 and 1.0, respectively. In Table 2, when $\omega_{vi}$ = 0.5, LRCFuse obtains five optimal values (En, SD, MI, SSIM and VIF). It can be concluded that in subsequent experiments, $\omega_{vi}$ is fixed at 0.5.

#### 4.1.3. The Impact of $L_{pixel}$ and $L_{deep}$

Next, the effects of $\gamma_{1}$ on $L_{pixel}$ and $\gamma_{4}$ on $L_{deep}$ are analyzed. The optimal value of $\gamma_{1}$ is defined within the interval {5,10,15,20}. The objective evaluation of different values of $\gamma_{1}$ is presented in Table 3.

When $\gamma_{1}=10$, the proposed fusion method obtains four optimal quality metrics, indicating that LRCFuse preserves both infrared features and details. Therefore, $\gamma_{1}$ is set to ten in the subsequent experiments. The ideal value of $\gamma_{4}$ is determined by sampling several values from the interval {700,1200,1700,2200}. The objective evaluations for different values of $\gamma_{4}$ are presented in Table 4. The fusion method identifies five optimal values and one suboptimal value when $\gamma_{4}=700$. Therefore, 700 is established as the optimal value for $\gamma_{4}$ in $L_{\mathrm{deep}}$.

#### 4.1.4. The Impact of $\gamma_{5}$ on $L_{Sobel}$

The optimal value of $\gamma_{5}$ is defined by sampling different values within the interval {100,1000,10,000}. The objective evaluations for different values of $\gamma_{5}$ are presented in Table 5. When $\gamma_{5}=1000$, the fusion method obtains three optimal values and two suboptimal values. Therefore, $\gamma_{5}$ in $L_{Sobel}$ is set to 1000.

### 4.2. Fusion Result Analysis

#### 4.2.1. Experiments on the TNO Dataset

**Figure 7:**
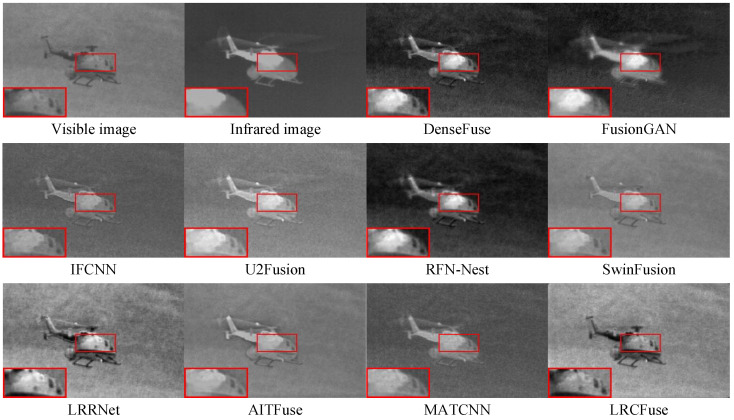
Fusion results of Helicopter in the TNO dataset. The red box shows the enlarged partial view of the figure.

Three pairs of representative images from the TNO dataset (named “Helicopter”, “Kaptein_1654”, and “Soldier_behind_smoke_1”) are chosen for qualitative assessment to visually illustrate the variations in fusion performance between LRCFuse and other methods. The fusion results are shown in Figure 7, in which the proposed method outperforms the other methods. For clear comparison, red boxes are used to select regions rich in textural information and yellow boxes are used to highlight areas with significant information in the images.

It can be observed from Figure 7 that the visible image detail information is partially injected into the fused image through DenseFuse, FusionGAN, and RFN-Nest, while the infrared target’s intensity is diminished. IFCNN, U2Fusion and MATCNN successfully maintain the salient objects in infrared images, although some textural are lost. SwinFusion, LRRNet, and AITFuse combine the complementary information from the original image, resulting in the blurred and unclear images. In contrast, the proposed LRCFuse effectively retains the rich textural details of visible images while maintaining the significant intensity of the targets.

**Figure 8:**
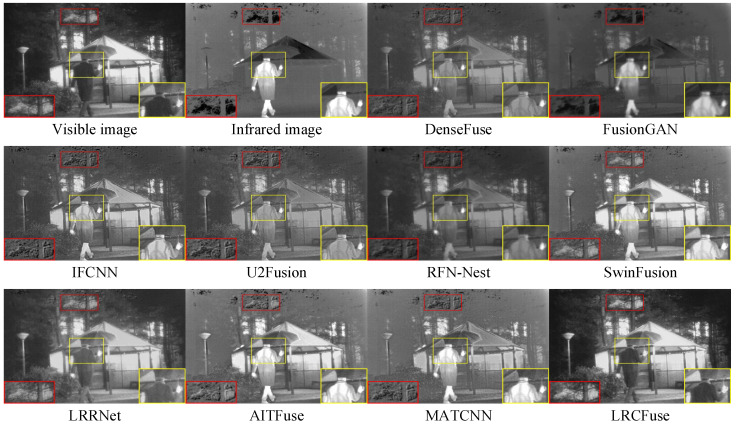
Fusion results of Kaptein_1654 in the TNO dataset. The red and yellow boxes show the enlarged partial view of the figure.

Figure 8 presents the visualization results, which may be broadly classified into two groups. The first type of results tend to be visible images, such as DenseFuse, IFCNN, U2Fusion, SwinFusion, and LRRNet. In particular, their fusion methods lose a considerable amount of the contrast information contained in infrared images, even as they retain certain textural features. The second type of images are closer to infrared images, including FusionGAN, RFN-Nest, AITFuse, and MATCNN. These methods effectively maintain significant contrast, but sacrifice depth in the textural details, resulting in images that resemble sharpened infrared images. In contrast, LRCFuse resembles more closely a hybrid of these two classifications, successfully highlighting the subject while preserving a sizable contrast and retaining rich texture structures in the fusion results.

**Figure 9:**
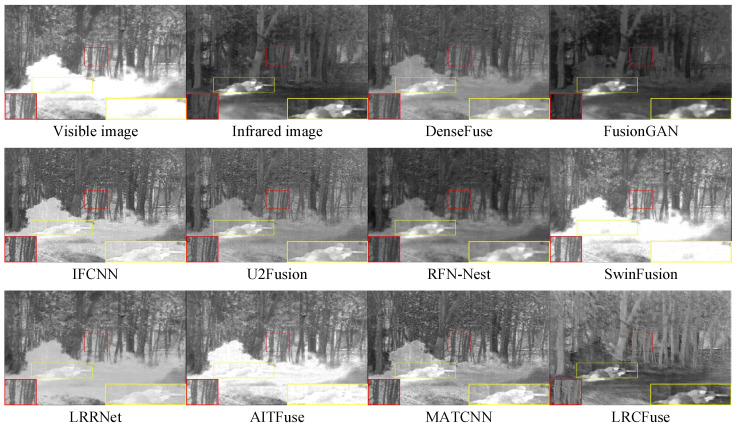
Fusion results of Soldier_behind_smoke_1 in the TNO dataset. The red and yellow boxes show the enlarged partial view of the figure.

For Figure 9, it can be observed that FusionGAN and AITFuse exhibit limitation in preserving textural and image clarity in their fusion results. IFCNN, U2Fusion, and SwinFusion appear blurry due to insufficient brightness to highlight the targets. Although DenseFuse, RFN-Nest and MATCNN can enhance the textural details of the targets, they are compromised by significant noise and a lack clarity in the fusion images. As opposed to the previously mentioned methods, LRCFuse effectively maintains the textural information apparent in visible images while also preserving sparse features from infrared images. Specifically, in the above images, the fuselage of the Helicopter achieves satisfactory infrared display without generating high-brightness highlights. In addition, the infrared features effectively contribute to the preservation and enhancement of the textural details of the targets.

Table 6 presents the average values of these metrics. Based on the average results, LRCFuse obtained the four optimal values (En, SD, MI, and VIF) with an increase of 1.12% for En, 4.01% for SD, 26.53% for MI, and 0.02% for VIF compared to the suboptimal values, while its Nabf dropped by 22.49%. Furthermore, according to the average ranking statistical analysis, our proposed model achieves the highest average ranking (Avg. Rank). Therefore, we can conclude that our method demonstrates a substantially superior comprehensive performance compared to the other approaches. This result indicates that the performance differences among the compared methods are statistically significant.

#### 4.2.2. Experiments on the RoadScene Dataset

**Figure 10:**
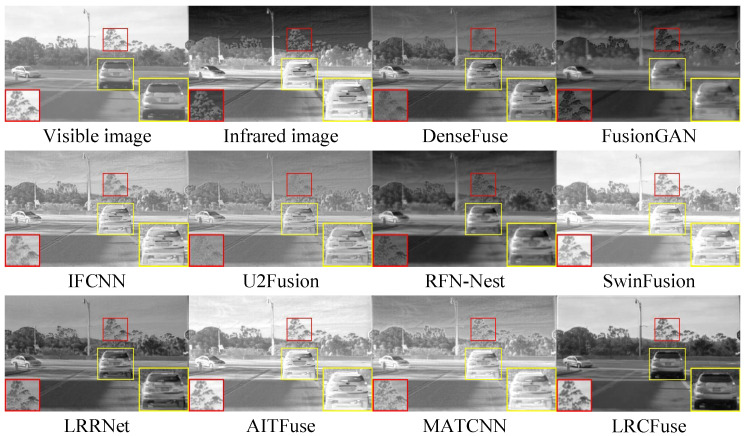
Fusion results of FLIR_00211 in the RoadScene dataset. The red and yellow boxes show the enlarged partial view of the figure.

**Figure 11:**
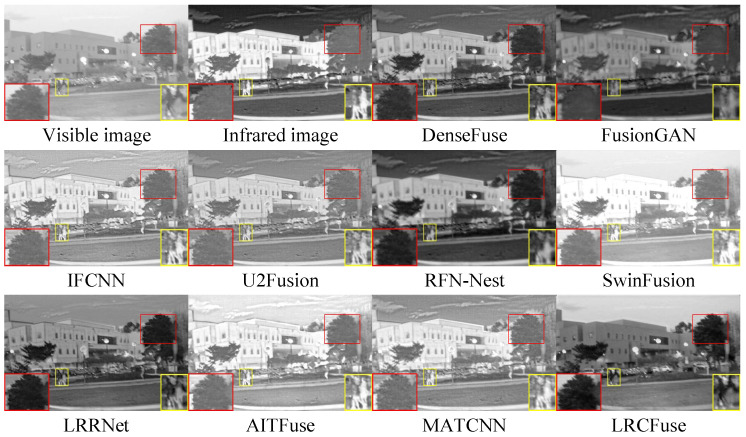
Fusion results of FLIR_00288 in the RoadScene dataset. The red and yellow boxes show the enlarged partial view of the figure.

**Figure 12:**
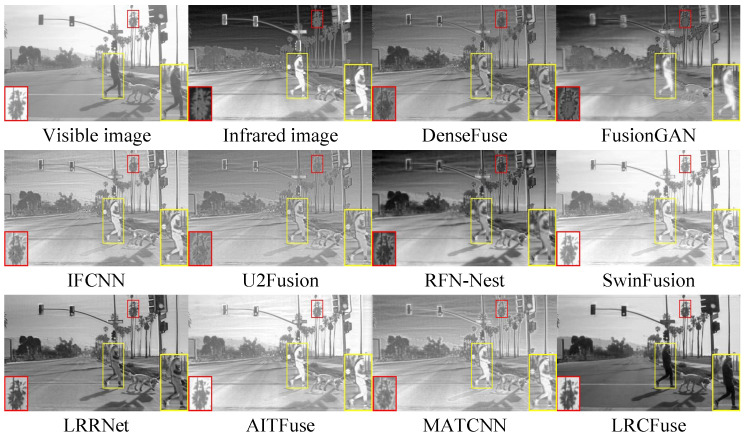
Fusion results of FLIR_04688 in the RoadScene dataset. The red and yellow boxes show the enlarged partial view of the figure.

Three pairs of representative images from the RoadScene dataset are selected for qualitative assessment (FLIR_00211, FLIR_00288 and FLIR_04688) (Figure 10, Figure 11 and Figure 12).

From the red box, it can be observed that the six models, except for SwinFusion, LRRNet, and MATCNN, exhibit relatively blurry textures on the tree branches and less clear car appearances due to high brightness levels. In contrast, the proposed LRCFuse provides clearer details in the tree branches and a more distinct car outline and surface compared to SwinFusion, LRRNet, and MATCNN. Additionally, Table 7 demonstrates that the fused images produced by LRCFuse are more consistent with human subjective perception and contain more detailed information.

On the RoadScene dataset, LRCFuse achieves four optimal values (En, SD, MI, and VIF), with increases of 1.11% for En, 29.12% for SD, 35.39% for MI, and 15.12% for VIF compared with the suboptimal results. Combined with the fact that LRCFuse obtained the highest Avg. Rank, the evidence strongly supports that our model provides a more effective and stable fusion strategy compared with other baselines. While LRCFuse leads in En, SD, MI, and VIF, its relatively higher $N_{abf}$ and lower SSIM reflect a calculated sharpness–contrast trade-off. Our deep unfolding mechanism intentionally intensifies edge gradients and local contrast to maximize information retention and thermal saliency (evidenced by the superior SD). However, because SSIM and $N_{abf}$ are sensitive to substantial deviations from source image brightness and structural distributions, they numerically penalize these enhancements as artifacts or structural shifts.

#### 4.2.3. Experiments on the M3FD Dataset

Three pairs of representative images from the M3FD dataset (00818, 02440 and 03708) are selected for qualitative assessment.

**Figure 13:**
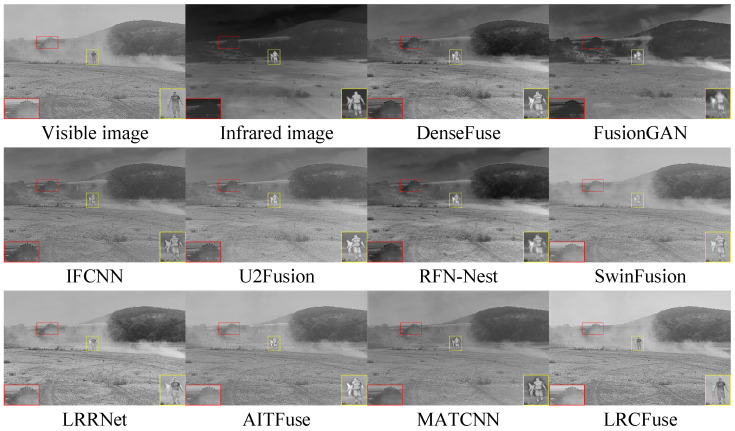
Fusion results of 00818 in the M3FD dataset. The red and yellow boxes show the enlarged partial view of the figure.

**Figure 14:**
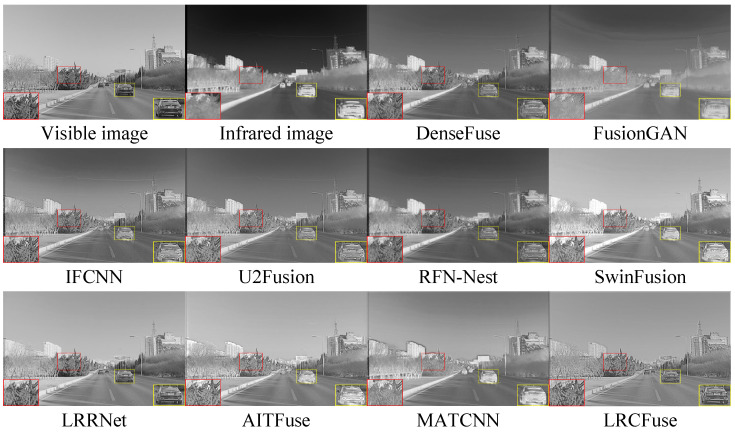
Fusion results of 02440 in the M3FD dataset. The red and yellow boxes show the enlarged partial view of the figure.

**Figure 15:**
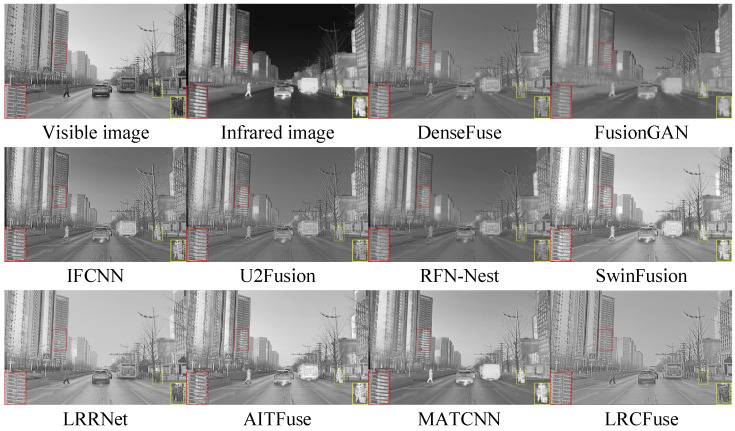
Fusion results of 03708 in the M3FD dataset. The red and yellow boxes show the enlarged partial view of the figure.

From Figure 13, Figure 14 and Figure 15, it can be observed that DenseFuse and IFCNN preserve some details, especially the contours of the targets, but the overall contrast of the images is relatively low. FusionGAN, U2Fusion, AITFuse, and MATCNN generate images that are overall clearer, but some regions have lower contrast. RFN-Nest, SwinFusion, and LRRNet produce images with good overall quality, but artifacts or distortions appear in certain areas. In comparison, the proposed LRCFuse performs exceptionally well in preserving the contours and textures of targets. It also handles detail retention, contrast, and noise processing effectively, resulting in clearer and more contrast-rich images.

From Table 8, it can be concluded that in the M3FD dataset, LRCFuse achieves the best values for En, SD, MI, and VIF, with increases of 1.41% for En, 22.63% for SD, 15.42% for MI, and 1.19% for VIF compared with the suboptimal results. The lower scores in $N_{abf}$ and SSIM highlight a clear trade-off between aggressive contrast enhancement and structural mimicry. To maximize target saliency, LRCFuse intentionally intensifies edge gradients and shifts local luminance distributions. While these modifications significantly improve visual clarity and downstream interpretability, they are numerically penalized by $N_{abf}$ as artifacts and by SSIM as structural deviations from the source images. This confirms that LRCFuse prioritizes informative feature recovery over conservative pixel-level similarity. Given that LRCFuse consistently secures the highest Avg. Rank, we can conclude that it offers the most competitive overall performance across the M3FD dataset. In addition, the proposed approach successfully maintains image quality while ensuring authenticity and naturalness.

### 4.3. Analysis of Computational Complexity

Table 9 presents a comprehensive comparison of various image fusion methods, including the proposed LRCFuse, in terms of model parameters, runtime, and computational complexity (FLOPs). To ensure a fair comparison, all the models were evaluated on the same hardware platform equipped with an Intel Core i7-3770K CPU, 32 GB of RAM, and an NVIDIA GeForce RTX 4090 GPU. Traditional lightweight CNN-based models such as DenseFuse and IFCNN maintain low parameter counts and FLOPs, yet still suffer from relatively long inference times. On the other hand, more complex networks like FusionGAN, U2Fusion, and RFN-Nest offer stronger representational power but exhibit significantly larger model sizes and computational overhead, which hinders their applicability in resource-constrained environments.

In contrast, LRCFuse achieves an optimal balance by requiring only 0.18 M parameters, with an inference time of 0.06 s and 171.64 GFLOPs. This demonstrates its exceptional memory efficiency with a compact architecture. While the FLOPs are relatively higher due to the iterative unfolding design, the minimal parameter count significantly reduces memory access costs, making the model particularly advantageous for memory-constrained deployment in real-time applications. Furthermore, in terms of training efficiency, LRCFuse requires only 0.74 h for convergence, achieving the second-best performance among all compared methods. Compared to heavy-duty models such as AITFuse (17.82 h), RFN-Nest (11.77 h), and MATCNN (10.76 h), which demand substantial temporal resources, LRCFuse significantly reduces the training overhead. The relatively short training duration, alongside its low inference latency, further validates the practical deployability and optimization efficiency of the proposed framework.

### 4.4. Ablation Experiments

#### 4.4.1. Effectiveness of LLRR-Blocks

To evaluate the effectiveness of the LLRR-Blocks, their quantity is incrementally increased from zero (no LLRR-Blocks) to four, and the resulting effects are demonstrated through objective evaluation presented in Table 10. The analysis reveals that with only one module, the model achieves a single optimal value for the Nabf. When three modules are employed, two optimal values for En and SD are obtianed. Furthermore, with four modules, the model achieves three optimal values for MI, SSIM, and VIF, with MI increasing by 10.96%, SSIM by 0.55%, and VIF by 10.11%. At this configuration, the model effectively leverages the rich information present in the original images.

#### 4.4.2. Effectiveness of CFPM-Blocks

Subsequently, in order to determine the validity of the modules adopted in the network architecture, an ablation experiment of the modules by removing or deleting some of them is conducted. As illustrated in Table 11, the introduction of CFPM-Blocks leads to improvements in four key metrics. The SD increases by 5.10%, MI rises by 42.98%, SSIM improves by 14.29%, and VIF enhances by 41.53%. Notably, MI and VIF exhibit the most significant gains. These findings demonstrate that the CFPM-Blocks clearly improve the contrast of fusion results, enhance retention of information from the original images, and contribute to more realistic outcomes.

#### 4.4.3. Effectiveness of the Loss Function

**Figure 16:**
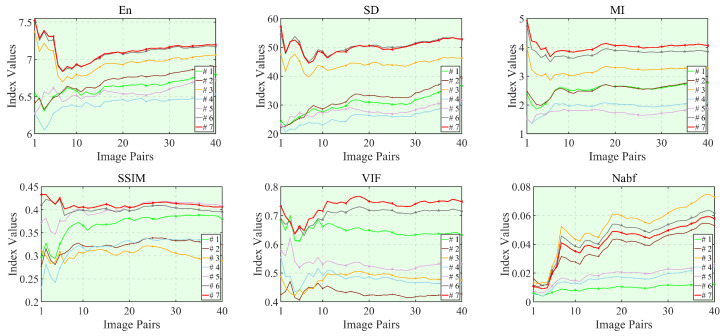
Objective evaluation results of the six metrics for the seven options in the ablation experiments.

$L_{pixel}$ influences whether the fused image can retain more detailed information, while $L_{shallow}$, $L_{middle}$, and $L_{deep}$ control the shallow, middle, and deep features of the fused image, respectively. Additionally, $L_{sobel}$ emphasizes the prominent infrared features in the fused image. Each component is individually set to zero to eliminate its impact, allowing for an analysis of effectiveness through the observation of changes in objective indicators. The specific objective evaluations are presented in Table 12. From Table 12, it can be observed that when only $L_{pixel}$ and $L_{middle}$ are included in the loss function, the fusion result obtains the lowest Nabf, suggesting that the model introduces the least artifacts during the fusion process. When the loss function is composed of $L_{pixel}$, $L_{shallow}$, $L_{middle}$ and $L_{deep}$, LRCFuse obtains the optimal values for En and SD, indicating that the spatial structure of the fusion result generated is the most consistent with the original image. Finally, when the loss function is fully composed of the five proposed terms, the best three indicators (MI, SSIM, and VIF) are obtained. Compared to the suboptimal value, the MI increases by 11.68%, SSIM by 1.24% and VIF by 11.05%. The same result is shown in Figure 16. This demonstrates that the fused images significantly enhance richness of the original images. In conclusion, the effectiveness of the proposed multi-level optimization is substantiated by the aforementioned results.

**Figure 17:**
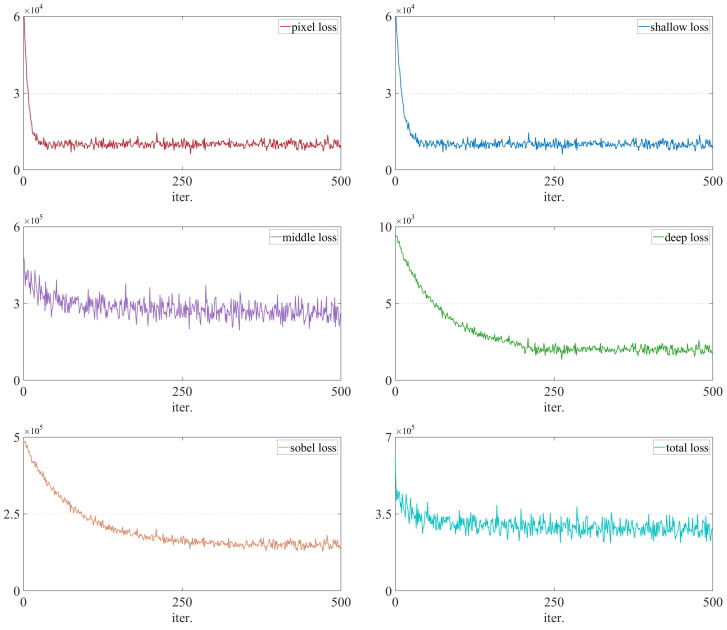
The loss values for each item of the proposed loss function with the selected optimal hyperparameters. For the lateral axis, each number indicates ten iterations in training phase.

Based on the previous discussion, the loss values for each part and the total loss are shown in Figure 17. As illustrated in Figure 17, $L_{pixel}$ and $L_{shallow}$ exhibit near-instantaneous convergence within 200 iterations, enabling the model to rapidly prioritize low-level textural details. While $L_{middle}$ displays inherent fluctuations—stemming from the competitive balancing between visible spatial information and infrared salient features—it reaches a steady state alongside the smooth, monotonic descent of $L_{sobel}$ and $L_{total}$. Overall, the network achieves a robust equilibrium, ensuring a harmonious trade-off between structural preservation and multi-modal feature enhancement.

## 5. Conclusions

This paper introduces a novel cross-modal image fusion approach, termed LRCFuse, which integrates LLRR-Blocks with CFPM. The LLRR module employs four convolutional layers to separately extract low-rank and sparse features from visible and infrared images. These features are concatenated and further processed through two additional convolution operations to produce the corresponding fused representations. In the CFPM module, an iterative algorithm is utilized to identify the shared features between the two modalities. The three resulting feature sets are then fused to generate the final output image. Moreover, a multi-level optimization is proposed to supervise the training process, enabling the model to effectively learn and refine cross-modal feature representations. The experimental results on two widely used infrared and visible image datasets, TNO and RoadScene, demonstrate that LRCFuse achieves significant improvements in the MI index over existing fusion techniques, with gains of 26.53% on TNO, 35.39% on RoadScene, and 15.42% on the M3FD dataset. Benefiting from its ability to preserve complementary structures and extract modality-common information, LRCFuse exhibits potential in multi-sensor image fusion tasks.

For future work, we aim to further reduce the model size through architectural simplifications and compression techniques, enhancing the deployment capability of LRCFuse on edge devices. While recent advancements such as Diffusion models and Mamba-based architectures have shown great potential in generative modeling and long-range dependency capture, our LRCFuse offers a more lightweight and interpretable alternative. Future research will explore integrating the stochastic priors of Diffusion models or the efficient scaling of Mamba into our deep unfolding framework to further enhance fusion quality while maintaining deployment efficiency. Additionally, extending LRCFuse to multi-scenario computational imaging, such as daytime and nighttime intelligent perception, will further expand its applicability in real-world systems.

## Figures and Tables

**Table 1:** The average values of the six objective metrics obtained with different parameters ($\gamma_{2}$ and $\omega_{ir}$) on the TNO dataset. The top two values are marked in bold and $\underline{underline}$. The upward or downward arrow indicates that the corresponding indicator is better when larger or smaller.

$\gamma_{2}$	$\omega_{\mathrm{ir}}$	En ↑	SD ↑	MI ↑	SSIM ↑	VIF ↑	Nabf ↓
1.3	1.0	6.75919	32.01696	1.95494	**0.42836**	0.56137	**0.02482**
1.9	1.0	**7.05884**	**50.52916**	**4.30793**	0.41257	**0.79406**	0.05712
1.3	2.0	6.85898	35.71782	2.16799	0.34504	0.52310	0.02812
1.9	2.0	7.02234	48.42461	3.52736	0.38297	0.61722	0.05483

**Table 2:** The average values of the six objective metrics obtained with different $\omega_{vi}$ on TNO dataset. The top two values are marked in bold and $\underline{underline}$. The upward or downward arrow indicates that the corresponding indicator is better when larger or smaller.

$\omega_{\mathrm{vi}}$	En ↑	SD ↑	MI ↑	SSIM ↑	VIF ↑	Nabf ↓
0.1	6.97363	49.71778	3.02953	0.33065	0.64395	0.07093
0.3	6.75330	37.74942	2.53364	0.38898	0.69493	**0.03096**
0.5	**7.05625**	**50.77412**	**4.52581**	**0.42338**	**0.83638**	0.06584
0.7	7.01546	45.35038	2.96784	0.36338	0.70242	0.06221

**Table 3:** The average values of the six objective metrics obtained with different $\gamma_{1}$ on TNO dataset. The top two values are marked in bold and $\underline{underline}$. The upward or downward arrow indicates that the corresponding indicator is better when larger or smaller.

$\gamma_{1}$	En ↑	SD ↑	MI ↑	SSIM ↑	VIF ↑	Nabf ↓
5	6.82935	38.75299	3.02835	0.40001	0.54827	0.02555
10	**7.05884**	**50.52916**	**4.30793**	0.41257	**0.79406**	0.05712
15	6.84087	39.36511	3.16580	0.41735	0.63104	**0.01695**
20	6.88275	38.06958	2.87119	**0.42676**	0.74371	0.01850

**Table 4:** The average values of the six objective metrics obtained with different $\gamma_{4}$ on TNO dataset. The top two values are marked in bold and $\underline{underline}$. The upward or downward arrow indicates that the corresponding indicator is better when larger or smaller.

$\gamma_{4}$	En ↑	SD ↑	MI ↑	SSIM ↑	VIF ↑	Nabf ↓
700	**7.05884**	**50.52916**	**4.30793**	**0.41257**	**0.79406**	0.05712
1200	6.85122	38.26567	2.69821	0.32403	0.39427	0.05794
1700	7.00957	42.12164	2.77548	0.32562	0.43852	0.05795
2200	6.72380	37.87535	4.01538	0.41243	0.75113	**0.01813**

**Table 5:** The average values of the six objective metrics obtained with different $\gamma_{5}$ on TNO dataset. The top two values are marked in bold and $\underline{underline}$. The upward or downward arrow indicates that the corresponding indicator is better when larger or smaller.

$\gamma_{5}$	En ↑	SD ↑	MI ↑	SSIM ↑	VIF ↑	Nabf ↓
10	**7.10008**	47.98404	3.39365	0.41143	0.70984	0.05543
100	6.96350	36.66765	2.13863	**0.42214**	0.59849	0.04076
1000	7.05884	**50.52916**	**4.30793**	0.41257	**0.79406**	0.05712
10,000	6.69025	30.88287	1.87072	0.38718	0.46414	**0.02681**

**Table 6:** The average values of the objective metrics obtained by the existing fusion methods and the proposed LRCFuse on TNO dataset. The top two values are marked in bold and $\underline{underline}$. The upward or downward arrow indicates that the corresponding indicator is better when larger or smaller.

Methods	En ↑	SD ↑	MI ↑	SSIM ↑	VIF ↑	Nabf ↓	Avg. Rank ↓
DenseFuse [18]	6.79183	34.31336	2.00681	0.49082	0.62504	0.02834	6.500
FusionGAN [22]	6.55022	30.04277	2.39626	0.28748	0.39798	0.07428	9.333
IFCNN [19]	6.71453	35.59594	1.98537	0.50411	0.62779	**0.01481**	5.833
U2Fusion [23]	6.66365	30.25378	1.80841	0.50631	0.60504	0.01639	6.667
RFN-Nest [30]	6.85937	36.10409	2.01709	0.39258	0.56991	0.07166	7.333
SwinFusion [25]	6.77242	38.54927	3.48708	0.50587	0.75525	0.03175	4.167
LRRNet [38]	6.96234	48.82049	3.15323	0.42704	0.64712	0.05538	4.500
AITFuse [32]	6.85840	38.09585	3.57696	0.47120	0.83619	0.02375	3.833
MATCNN [33]	6.97591	38.19316	2.84512	**0.50872**	0.64068	0.02754	3.500
LRCFuse	**7.05625**	**50.77412**	**4.52581**	0.42338	**0.83638**	0.06584	**3.333**

**Table 7:** The average values of the objective metrics obtained by the existing fusion methods and the proposed LRCFuse on RoadScene dataset. The top two values are marked in bold and $\underline{underline}$. The upward or downward arrow indicates that the corresponding indicator is better when larger or smaller.

Methods	En ↑	SD ↑	MI ↑	SSIM ↑	VIF ↑	Nabf ↓	Avg. Rank ↓
DenseFuse [18]	7.09109	36.81824	2.51636	0.47759	0.51573	0.07515	6.333
FusionGAN [22]	7.05175	37.15094	2.52492	0.28513	0.33586	0.14690	8.833
IFCNN [19]	7.16352	38.16060	2.58084	0.47516	0.52472	**0.03803**	4.667
U2Fusion [23]	6.90374	32.16385	2.37272	0.48445	0.48872	0.05036	6.833
RFN-Nest [30]	7.22709	40.92221	2.37572	0.36541	0.43719	0.14547	7.167
SwinFusion [25]	6.92465	42.00547	3.30714	0.46661	0.57424	0.08701	5.000
LRRNet [38]	7.12928	41.98335	2.69499	0.32662	0.44614	0.10459	6.667
AITFuse [32]	7.21155	43.75576	3.22339	0.43396	0.68660	0.05117	3.500
MATCNN [33]	7.24457	43.91766	2.51151	**0.50128**	0.59896	0.09409	3.667
LRCFuse	**7.32496**	**56.71038**	**4.47762**	0.33426	**0.79044**	0.11093	**3.333**

**Table 8:** The average values of the objective metrics obtained by the existing fusion methods and the proposed LRCFuse on M3FD dataset. The top two values are marked in bold and $\underline{underline}$. The upward or downward arrow indicates that the corresponding indicator is better when larger or smaller.

Methods	En ↑	SD ↑	MI ↑	SSIM ↑	VIF ↑	Nabf ↓	Avg. Rank ↓
DenseFuse [18]	6.58909	28.02175	2.92549	0.43890	0.60585	0.01603	7.333
FusionGAN [22]	6.84747	34.08980	3.37332	0.31365	0.37809	0.02371	6.333
IFCNN [19]	6.96057	34.01236	2.67488	0.41917	0.65113	**0.00777**	6.667
U2Fusion [23]	6.81388	30.90184	2.82955	0.43907	0.60289	0.00831	7.333
RFN-Nest [30]	6.73512	30.64019	2.93755	0.35666	0.54032	0.02300	7.333
SwinFusion [25]	6.84366	35.36096	4.40966	**0.46684**	0.66574	0.01376	3.500
LRRNet [38]	6.76404	36.79772	3.81496	0.35461	0.56362	0.01943	5.667
AITFuse [32]	6.87806	35.09433	4.21535	0.44118	0.73810	0.01177	3.667
MATCNN [33]	6.86193	34.60871	3.37391	0.45410	0.65620	0.00899	4.667
LRCFuse	**7.05881**	**45.12678**	**5.09003**	0.39069	**0.75435**	0.01727	**2.500**

**Table 9:** Average test time, training time and parameters of different methods. The top two values are marked in bold and $\underline{underline}$.

Methods	Training Time (h)	Parameter (M)	Runtime (s)	FLOPS (G)
DenseFuse [18]	1.42	0.30	0.28	48.96
FusionGAN [22]	—	0.93	0.66	497.76
IFCNN [19]	—	0.08	0.71	**40.04**
U2Fusion [23]	0.78	0.66	0.97	366.34
RFN-Nest [30]	11.77	7.52	0.36	520.80
SwinFusion [25]	1.31	0.97	0.58	307.51
LRRNet [38]	**0.42**	**0.04**	**0.02**	113.56
ITFuse [32]	17.82	6.89	0.13	470.13
MATCNN [33]	10.76	5.76	0.61	188.82
LRCFuse	0.74	0.18	0.06	171.64

**Table 10:** The average values of the objective metrics obtained on TNO dataset with gradually increasing LLRR-Blocks. The top two values are marked in bold and $\underline{underline}$. The upward or downward arrow indicates that the corresponding indicator is better when larger or smaller.

Experiments	En ↑	SD ↑	MI ↑	SSIM ↑	VIF ↑	Nabf ↓
Block = 0	6.72048	37.85642	2.73412	0.37894	0.50237	0.05845
Block = 1	6.89916	41.10119	3.16853	0.40532	0.58458	**0.02656**
Block = 2	7.11842	47.98028	3.54054	0.41029	0.72112	0.05592
Block = 3	**7.14171**	**52.43695**	3.88244	0.39002	0.71973	0.05772
Block = 4	7.05884	50.52916	**4.30793**	**0.41257**	**0.79406**	0.05712

**Table 11:** The average values of the objective metrics obtained on TNO dataset with or without CFPM-Blocks. The top value is marked in bold. The upward or downward arrow indicates that the corresponding indicator is better when larger or smaller.

Experiments	En ↑	SD ↑	MI ↑	SSIM ↑	VIF ↑	Nabf ↓
without CFPM	7.15041	48.07514	3.01300	0.36099	0.56104	**0.05603**
with CFPM	7.05884	**50.52916**	**4.30793**	**0.41257**	**0.79406**	0.05712

**Table 12:** The average values of the objective metrics obtained on TNO dataset with or without loss function. The top two values are marked in bold and $\underline{underline}$. The # represents numbers corresponding to different combinations of parameters. The upward and downward arrows indicate that larger or smaller values of the indicator are better.

$\gamma_{1}$	$\gamma_{2}$	$\gamma_{4}$	$\gamma_{5}$	#	En ↑	SD ↑	MI ↑	SSIM ↑	VIF ↑	Nabf ↓
✔				1	6.79569	36.66344	2.76832	0.37946	0.63172	**0.01222**
	✔			2	6.90196	39.28867	2.79392	0.32487	0.42684	0.05295
		✔		3	7.06123	46.30870	3.28099	0.28924	0.47584	0.07267
			✔	4	6.53447	30.29180	2.11628	0.32778	0.46019	0.02398
✔	✔			5	6.72465	31.66982	1.87771	0.40751	0.53230	0.02577
✔	✔	✔		6	**7.11799**	**52.96718**	3.85717	0.39593	0.71505	0.06182
✔	✔	✔	✔	7	7.05884	50.52916	**4.30793**	**0.41257**	**0.79406**	0.05712

## Data Availability

Publicly available datasets were analyzed in this study. This data can be found here: TNO at https://github.com/yanyanchun/TNO_Image_Fusion_Dataset, accessed on 5 March 2026; RoadScene at https://github.com/hanna-xu/RoadScene, accessed on 5 March 2026; and M3FD at https://github.com/JinyuanLiu-CV/TarDAL, accessed on 5 March 2026.
